# Supplementary material for: Parental behavior, adult attachment, and DNA methylation of the MT2 oxytocin receptor gene region – The moderating role of neuroticism
Source: PLoS One. 2026 Feb 20;21(2):e0341072. doi: 10.1371/journal.pone.0341072 (PMC12923032; doi:10.1371/journal.pone.0341072)
Supplement: S1 Table — Maternal care and attachment anxiety: mediated by MT2 methylation levels and moderated by neuroticism. (DOCX) [file pone.0341072.s001.docx]

**S1 Table. Results of the moderated mediation in hypothesis b.**Maternal care and attachment anxiety: mediated by MT2 methylation levels and moderated by neuroticism.

| **Predictor** | **Attachment Anxiety** | | | |  | **OXTR MT2 Mean Methylation Rates** | | | | |
| --- | --- | --- | --- | --- | --- | --- | --- | --- | --- | --- |
|  | ***b*** | **SE (HC3)** | **LLCI** | **ULCI** | | | ***b*** | **SE (HC3)** | **LLCI** | **ULCI** |
| **Constant** | 3.269*** | 0.718 | 1.836 | 4.702 | | | 81.050*** | 0.872 | 79.309 | 82.790 |
| **Maternal Care** | 0.015 | 0.012 | -0.009 | 0.038 | | | -0.320 | 0.185 | -0.688 | 0.049 |
| **Neuroticism Scores** | 0.818*** | 0.115 | 0.589 | 1.047 | | | -0.515 | 1.553 | -3.616 | 2.585 |
| **OXTR MT2 Methylation** | 0.005 | 0.009 | -0.013 | 0.022 | | |  |  |  |  |
| **Maternal Care x Neuroticism** | -0.031 | 0.023 | -0.077 | 0.016 | | | 0.896* | 0.388 | 0.121 | 1.670 |
| ***R²*** | .452*** |  |  |  | | | .118* |  |  |  |

*Note:* Standardized regression coefficients are reported. Listwise *N* = 71, SE (HC3) = Davidson-MacKinnon standard error; LLCI = lower-level confidence interval; ULCI = upper-level confidence interval, Bootstrap sample size = 5000; confidence interval 95%; **p* < .05 ****p* < .001
