# Supplementary material for: Parental behavior, adult attachment, and DNA methylation of the MT2 oxytocin receptor gene region – The moderating role of neuroticism
Source: PLoS One. 2026 Feb 20;21(2):e0341072. doi: 10.1371/journal.pone.0341072 (PMC12923032; doi:10.1371/journal.pone.0341072)
Supplement: S2 Table — Paternal care and attachment avoidance: mediated by MT2 methylation levels and moderated by neuroticism. (DOCX) [file pone.0341072.s002.docx]

**S2 Table. Results of the moderated mediation in hypothesis c.**Paternal care and attachment avoidance: mediated by MT2 methylation levels and moderated by neuroticism.

| **Predictor** | **Attachment Avoidance** | | | |  | **OXTR MT2 Mean Methylation Rates** | | | | |
| --- | --- | --- | --- | --- | --- | --- | --- | --- | --- | --- |
|  | ***b*** | **SE (HC3)** | **LLCI** | **ULCI** | | | ***b*** | **SE (HC3)** | **LLCI** | **ULCI** |
| **Constant** | 1.645* | 0.671 | 0.304 | 2.985 | | | 80.366*** | 0.890 | 78.589 | 82.144 |
| **Paternal Care** | -0.009 | 0.007 | -0.024 | 0.006 | | | -0.083 | 0.110 | -0.303 | 0.138 |
| **Neuroticism Scores** | 0.497*** | 0.120 | 0.259 | 0.736 | | | 1.004 | 1.541 | -2.071 | 4.079 |
| **OXTR MT2 Methylation** | 0.014 | 0.008 | -0.002 | 0.031 | | |  |  |  |  |
| **Paternal Care x Neuroticism** | 0.002 | 0.012 | -0.023 | 0.026 | | | 0.083 | 0.174 | -0.264 | 0.429 |
| ***R²*** | .332*** |  |  |  | | | .021 |  |  |  |

*Note:* Standardized regression coefficients are reported. Listwise *N* = 71, SE (HC3) = Davidson-MacKinnon standard error; LLCI = lower-level confidence interval; ULCI = upper-level confidence interval, Bootstrap sample size = 5000; confidence interval 95%; **p* < .05 ****p* < .001
