# Supplementary material for: Parental behavior, adult attachment, and DNA methylation of the MT2 oxytocin receptor gene region – The moderating role of neuroticism
Source: PLoS One. 2026 Feb 20;21(2):e0341072. doi: 10.1371/journal.pone.0341072 (PMC12923032; doi:10.1371/journal.pone.0341072)
Supplement: S3 Table — Paternal care and attachment anxiety: mediated by MT2 methylation levels and moderated by neuroticism. (DOCX) [file pone.0341072.s003.docx]

**S3 Table. Results of the moderated mediation in hypothesis d.**Paternal care and attachment anxiety: mediated by MT2 methylation levels and moderated by neuroticism.

| **Predictor** | **Attachment Anxiety** | | | |  | **OXTR MT2 Mean Methylation Rates** | | | | |
| --- | --- | --- | --- | --- | --- | --- | --- | --- | --- | --- |
|  | ***b*** | **SE (HC3)** | **LLCI** | **ULCI** | | | ***b*** | **SE (HC3)** | **LLCI** | **ULCI** |
| **Constant** | 3.645*** | 0.731 | 2.185 | 5.106 | | | 80.366*** | 0.890 | 78.589 | 82.144 |
| **Paternal Care** | -0.006 | 0.009 | -0.023 | 0.012 | | | -0.083 | 0.110 | -0.303 | 0.138 |
| **Neuroticism Scores** | 0.723*** | 0.012 | 0.473 | 0.970 | | | 1.003 | 1.541 | -2.071 | 4.079 |
| **OXTR MT2 Methylation** | 0.001 | 0.009 | -0.017 | 0.018 | | |  |  |  |  |
| **Paternal Care x Neuroticism** | -0.003 | 0.012 | -0.028 | 0.021 | | | 0.083 | 0.174 | -0.264 | 0.429 |
| ***R²*** | .440*** |  |  |  | | | .021 |  |  |  |

*Note:* Standardized regression coefficients are reported. Listwise *N* = 71, SE (HC3) = Davidson-MacKinnon standard error; LLCI = lower-level confidence interval; ULCI = upper-level confidence interval, Bootstrap sample size = 5000; confidence interval 95%; ****p* < .001
