# Supplementary material for: Parental behavior, adult attachment, and DNA methylation of the MT2 oxytocin receptor gene region – The moderating role of neuroticism
Source: PLoS One. 2026 Feb 20;21(2):e0341072. doi: 10.1371/journal.pone.0341072 (PMC12923032; doi:10.1371/journal.pone.0341072)
Supplement: S5 Table — Mediated by MT2 methylation levels and moderated by neuroticism. (DOCX) [file pone.0341072.s005.docx]

**S5 Table. Results of the moderated mediation for maternal care and Relationships as Secondary.**Mediated by MT2 methylation levels and moderated by neuroticism,

| **Predictor** | **Discomfort with Closeness** | | | |  | **OXTR MT2 Mean Methylation Rates** | | | | |
| --- | --- | --- | --- | --- | --- | --- | --- | --- | --- | --- |
|  | ***b*** | **SE (HC3)** | **LLCI** | **ULCI** | | | ***b*** | **SE (HC3)** | **LLCI** | **ULCI** |
| **Constant** | 1.903* | 0.729 | 0.448 | 3.360 | | | 81.050*** | 0.872 | 79.309 | 82.790 |
| **Maternal Care** | -0.023* | 0.011 | -0.046 | -0.001 | | | -0.320 | 0.185 | -0.688 | 0.049 |
| **Neuroticism Scores** | 0.246 | 0.106 | 0.033 | 0.458 | | | -0.515 | 1.553 | -3.616 | 2.585 |
| **OXTR MT2 Methylation** | 0.002 | 0.009 | -0.016 | 0.020 | | |  |  |  |  |
| **Maternal Care x Neuroticism** | 0.017 | 0.018 | -0.018 | 0.052 | | | 0.896* | 0.388 | 0.121 | 1.670 |
| ***R²*** | .154** |  |  |  | | | .118* |  |  |  |

*Note:* Standardized regression coefficients are reported. Listwise *N* = 71, SE (HC3) = Davidson-MacKinnon standard error; LLCI = lower-level confidence interval; ULCI = upper-level confidence interval, Bootstrap sample size = 5000; confidence interval 95%; **p* < .05 ***p* < .01 ****p* < .001
