# Supplementary material for: Parental behavior, adult attachment, and DNA methylation of the MT2 oxytocin receptor gene region – The moderating role of neuroticism
Source: PLoS One. 2026 Feb 20;21(2):e0341072. doi: 10.1371/journal.pone.0341072 (PMC12923032; doi:10.1371/journal.pone.0341072)
Supplement: S6 Table — Bivariate correlation analysis for a subset of samples (n = 9) re-analyzed with a four-month gap in between the analyses. (DOCX) [file pone.0341072.s006.docx]

**S6 Table. Method check.**Bivariate correlation analysis for a subset of samples (*n* = 9) re-analyzed with a four-month gap in between the analyses.

| **Predictor** | **Methyl_3 at time-point 1 [%]** | | **Methyl_3 at time-point 2 [%]** | | **Correlation coefficient** | | **Significance** | |
| --- | --- | --- | --- | --- | --- | --- | --- | --- |
| **1** | | 80.32 | | 75.47 | | .924 | | *p* < .001 |
| **2** | | 74.90 | | 72.61 | | .924 | | *p* < .001 |
| **3** | | 77.42 | | 79.78 | | .966 | | *p* < .001 |
| **4** | | 84.51 | | 65.31 | | .897 | | *p* < .001 |
| **5** | | 42.51 | | 90.43 | | .754 | | *p* < .001 |
| **6** | | 24.55 | | 83.18 | | .705 | | *p* < .001 |
| **7** | | 82.56 | | 67.28 | | .913 | | *p* < .001 |
| **8** | | 77.50 | | 73.61 | | .925 | | *p* < .001 |
| **9** | | 85.96 | | 33.50 | | .394 | | *p* = 0.46 |
