# Supplementary material for: Parental behavior, adult attachment, and DNA methylation of the MT2 oxytocin receptor gene region – The moderating role of neuroticism
Source: PLoS One. 2026 Feb 20;21(2):e0341072. doi: 10.1371/journal.pone.0341072 (PMC12923032; doi:10.1371/journal.pone.0341072)
Supplement: Attachment — Submitted filename: Recommendation_Plos One.pdf [file pone.0341072.s007.pdf]

Dear Editor,

I express my thanks for choosing me as a reviewer of this article.

The manuscript tackles an important and timely question concerning the interplay between early parental behavior, DNA methylation of the oxytocin receptor gene (OXTR), and adult attachment, with neuroticism as a potential moderator. The integration of genetic, psychological, and environmental perspectives is both innovative and relevant to the field.

That said, the current version of the study reads more as an exploratory pilot than as a conclusive contribution. While the topic is promising, there are substantial concerns related to methodology, statistical rigor, and clarity of presentation. At present, the findings should be regarded as preliminary. Significant revisions are therefore necessary before the manuscript can be considered suitable for publication.

Introduction

1. The introduction is overly lengthy and, at times, redundant. Several sections reiterate similar content (e.g., general background on methylation and OXTR polymorphisms). A more concise and streamlined narrative would enhance readability.
2. The rationale for focusing on the MT2 region and the selected CpG sites should be articulated more clearly, with stronger justification.
3. The proposed mediating role of methylation is ambitious given the limited sample size and should be framed more cautiously. A more tempered tone is recommended.
4. The statement *"Parents, who described themselves as more securely attached, had children with more secure attachment styles, which supports the assumption of transgenerational transmission of attachment"* is overly direct. The concept of transgenerational transmission of attachment is considerably more complex and nuanced. A reformulation is advised, supported by authoritative references, given the importance of the issue.
5. The type of study conducted should be made more explicit (i.e., exploratory and preliminary in nature).

Methods

1. In the description of participant recruitment, it is stated that no exclusion criteria were applied. Typically, studies adopt screening procedures to exclude individuals reporting cognitive disabilities, intellectual impairments, or psychopathology. It would be important to clarify the rationale behind the decision not to apply any exclusion criteria in this case.
2. The final sample (N = 71) is very small compared to the original cohort (N = 367). While the reasons for exclusion (e.g., missing data, insufficient DNA) are described, the potential for systematic selection bias should be explicitly acknowledged in the limitations.
3. Statistical power is likely insufficient for the complex moderated mediation models employed. This limitation should be discussed.

4. The sample is homogeneous (psychology students, predominantly female, all Caucasian, mean age 21), which substantially restricts the generalizability of the findings. This important limitation should be emphasized.
5. The Parental Bonding Instrument (PBI) is a valid and widely used tool for assessing retrospective perceptions of parental care and control. However, given the age range of participants (some as old as 48), recall bias is a relevant concern that should be highlighted as a limitation.
6. Attachment was assessed exclusively through the self-report ASQ. Since more statistically robust instruments are available, it would be useful to justify the choice of this specific measure.
7. Relevant contextual factors (e.g., family structure, SES, life events) that could confound the observed associations were not collected. This omission should be addressed as a limitation.
8. The aggregation of the three CpG sites (-901, -924, -934) into a single composite index (Methyl_3) is not fully justified, especially given that intercorrelations among them were not particularly strong. The rationale for this analytical decision should be clarified.

Results

1. Bonferroni correction was applied only to selected hypotheses. The rationale for this selective application is unclear, and an explicit explanation should be provided.

Discussion

1. The discussion is overly long and, at times, digressive. A more concise and focused narrative would improve readability. For instance, certain sections (e.g., lines 454–461) deviate from the main topic and should be reconsidered.
2. While the discussion raises interesting points, it tends to justify null results with excessive speculation (e.g., small sample size, retrospective recall, genomic region differences). A more critical and balanced tone is recommended.
3. Causal language should be avoided, as the cross-sectional design does not allow for causal inference. The phrasing of several statements should be revised accordingly.
4. The role of paternal care is somewhat downplayed. The absence of significant effects should be acknowledged directly rather than extensively speculated upon.
5. The conclusion should be reframed: the findings provide exploratory evidence rather than definitive support for a mediation/moderation model.
6. Numerical results should generally not be repeated in the discussion, in order to facilitate readability.
7. It should be made explicit that the study is exploratory and preliminary in nature.
8. The practical significance of the findings should be discussed more clearly: what contribution do these data make to the field, and what are the implications for future research?

Conclusion

1. A more measured and cautious tone is recommended in the conclusions, in order to better reflect the exploratory and preliminary nature of the study.

Minor Revisions

1. While the manuscript is generally understandable, it would benefit from careful language editing. For example:
    a. “on how early experiences…” → should be “of how early experiences…”
    b. “became reimbursed” → should be “was reimbursed”
    c. “soley” → should be “solely”
2. Several sentences are overly long and difficult to follow; breaking them into shorter units would improve readability.
3. A professional English language review is strongly recommended.
4. The abstract should be revised to be shorter and more focused, highlighting only the key findings and limitations. In summary, improvements in clarity and conciseness are required.
