# Supplementary material for: Parental behavior, adult attachment, and DNA methylation of the MT2 oxytocin receptor gene region – The moderating role of neuroticism
Source: PLoS One. 2026 Feb 20;21(2):e0341072. doi: 10.1371/journal.pone.0341072 (PMC12923032; doi:10.1371/journal.pone.0341072)
Supplement: Attachment — Submitted filename: Response to Reviewers.docx [file pone.0341072.s009.docx]

| **Editor’s/Reviewers’ comments** | **Authors response** |
| --- | --- |
| **Editor** |  |
| We note that you have included the phrase “data not shown” in your manuscript. Unfortunately, this does not meet our data sharing requirements. PLOS does not permit references to inaccessible data. We require that authors provide all relevant data within the paper, Supporting Information files, or in an acceptable, public repository. Please add a citation to support this phrase or upload the data that corresponds with these findings to a stable repository (such as Figshare or Dryad) and provide and URLs, DOIs, or accession numbers that may be used to access these data. Or, if the data are not a core part of the research being presented in your study, we ask that you remove the phrase that refers to these data. | The data does not present a core part of the research; therefore, the phrase was removed. |
| **Reviewer 1** |  |
| Oversimplification of transgenerational attachment transmission | This is an important remark, thank you. The statement regarding transgenerational attachment transmission was modified and stated more cautiously: Feeney highlighted parallels to the model of intergenerational transmission by IJzendoorn & Bakermans-Kraneburg. However, this conclusion has to be drawn with caution, because the intergenerational transmission of attachment goes beyond the attachment pattern of the parents. Other factors, for example psychosocial risk factors or the age of the children, may vary the strength of the association, for a revised model see Verhage et al., 2016 [22] (line 119 to 126). |
| Significant generalizability concerns: participants were recruited from university settings without specified criteria, potentially including individuals with unreported psychiatric conditions (anxiety, depression, autism spectrum disorders) | We are aware of the problem raised. In fact, we included participants without exclusion criteria which might include a small number of those with affective disorders. In many studies we focused on that and did not find students with severe psychiatric disorders (schizophrenia, autism spectrum disorders, bipolar diseases etc.). Studies including neuroticism as one of the most important variables – as ours – have to solve the conflict between carefully select subjects on the one and reducing variance in neuroticism on the other hand. Consequently, it must be discussed whether samples excluding subjects high on neuroticism would meet the criterion of generalizability better than those including them. We discussed this at line 585 to 590: One point to consider is that we did not pre-screen our sample for any exclusion criteria, for example unreported psychiatric conditions. The main reason for that is our focus on neuroticism as a key variable in this study. Many psychiatric conditions, for example anxiety or depression, are linked to neuroticism [68]. Our goal was to have as much variance in neuroticism as possible. Additionally, excluding participants with high neuroticism scores would limit generalizability, since it does not represent the general population. |
| Wide age range as potential confounding variable | This addresses an important point. However, there was only one participant who was 48 years of age, the other participants were between 18 and 28 years of age. Since the PBI is well-established over a 20-year period, the majority of our sample falls into this age. Because of the already small sample size, we decided not to exclude the 48-year-old participant (line 640 to 635). |
| Lack of information about participants' mothers' childhood experiences, attachment histories, and maternal methylation patterns as crucial missing context for interpreting offspring outcomes. | This is an important comment. Due to the design of the initial study, we did not have this information. However, we included this point into future directions in line 612 to 614 (to support this assumption, future studies should collect more information about mothers (or the primary caregiver being studied), for example childhood experiences, life-events, or attachment style) and in line 649 to 652 (A bigger sample also provides the chance to include primary caregivers divided into certain attachment styles, to further elaborate on the theory of intergenerational transmission. In order to do that the primary caregiver’s childhood experiences, methylation status and attachment styles should be collected). |
| More cautious framing of the results: interesting but preliminary | This crucial point was repeatedly mentioned by the reviewers. We considered this mainly in the results section and the discussion. |
| Suggestions for Future Directions: Continue longitudinal follow-up with more rigorous clinical characterization. Investigate relationship between neuroticism and temperamental inhibition (RGS2 gene), particularly regarding social anxiety phenotypes. Recruit more targeted samples (e.g., offspring of mothers with documented childhood trauma and known oxytocin receptor methylation patterns) to test specific hypotheses about intergenerational transmission | The part about the more targeted sample was included in the text: To support this assumption, future studies should collect more information about mothers (or the primary caregiver being studied), for example childhood experiences, life-events, or attachment style.Additionally, longitudinal studies are recommended to detect changes in attachment over the life-span (line 612-615). We decided not to include the reference to the RGS2 gene, because our focus is on oxytocin and a convenience sample, rather than social anxiety phenotypes. |
| More measured and cautious tone to reflect the exploratory and preliminary nature of the study | This crucial point was repeatedly mentioned by the reviewers. We considered this mainly in the results section and the discussion. |
| **Reviewer 2** |  |
| The introduction is overly lengthy and, at times, redundant. Several sections reiterate similar content (e.g., general background on methylation and OXTR polymorphisms). A more concise and streamlined narrative would enhance readability. | The introduction follows the following logic: at first information about oxytocin in general where presented, followed by general information about DNA methylation. Then methylation of the OXTR is discussed. Afterwards, the second topic (attachment) is introduced, next, the personality factor neuroticism. Leading to the hypotheses. Some parts of the introduction were clarified and shortened; however, the original structure remained the same. |
| The rationale for focusing on the MT2 region and the selected CpG sites should be articulated more clearly, with stronger justification. | This is a valid question. When it comes to epigenetic studies regarding oxytocin, the MT2 region is the most prominent, because its functional relevance has been shown in several studies, most important Kusui et al. and Danoff et al. Especially Danoff showed that the selected CpG sites are related to early life experiences, which makes them suitable for our study (line 77 to 78 and 84 to 86). |
| The proposed mediating role of methylation is ambitious given the limited sample size and should be framed more cautiously. A more tempered tone is recommended. | This crucial point was repeatedly mentioned by the reviewers. We considered this mainly in the results section and the discussion. Additionally, the hypotheses were framed more cautiously: If perceived parental care in childhood is low, this may be accompanied by changes in OXTR MT2 methylation levels. As a consequence, avoidant and anxious attachment may increase. Therefore, it is assumed that the relationship between perceived parental care and attachment is associated with MT2 methylation levels (line 172 to 175) and: Higher levels of perceived care in childhood are associated with decreased levels of [a] attachment avoidance and [b] attachment anxiety, both being associated with methylation rates in the OXTR MT2 region. (line 184 to 186). |
| The statement *“Parents, who described themselves as more securely attached, had children with more secure attachment styles, which supports the assumption of transgenerational transmission of attachment”* is overly direct. The concept of transgenerational transmission of attachment is considerably more complex and nuanced. A reformulation is advised, supported by authoritative references, given the importance of the issue. | This is an important remark, thank you. The statement regarding transgenerational attachment transmission was modified and stated more cautiously: Feeney highlighted parallels to the model of intergenerational transmission by IJzendoorn & Bakermans-Kraneburg. However, this conclusion has to be drawn with caution, because the intergenerational transmission of attachment goes beyond the attachment pattern of the parents. Other factors, for example psychosocial risk factors or the age of the children, may vary the strength of the association, for a revised model see Verhage et al., 2016 [22] (line 119 to 126). |
| The type of study conducted should be made more explicit (i.e., exploratory and preliminary in nature). | A really important consideration, which was added in line 166 to 168: In line with the model of gene x environment interactions, the present study, exploratory and preliminary in nature, examines the association between parental behavior, MT2 methylation, and attachment styles later in life. |
| In the description of participant recruitment, it is stated that no exclusion criteria were applied. Typically, studies adopt screening procedures to exclude individuals reporting cognitive disabilities, intellectual impairments, or psychopathology. It would be important to clarify the rationale behind the decision not to apply any exclusion criteria in this case. | Again, in German Universities enrollment in the training of Psychology requires many proofs of high intellectual performance. Therefore, cognitive and/or mental disabilities can be ruled out. Severe psychopathology (see above) is usually combined with absence from University courses. Affective Disorders may be included in a very small number of participants. This was accepted in order to gain a broad range in neuroticism. A justification is now included at line 585 to 590 (see above). |
| The final sample (N = 71) is very small compared to the original cohort (N = 367). While the reasons for exclusion (e.g., missing data, insufficient DNA) are described, the potential for systematic selection bias should be explicitly acknowledged in the limitations. | The reviewer addresses an important point and a shortcoming of the present study. We discussed the large dropout in line 664 to 670: The large drop-out, the initial sample had *N* = 367 participants, whereas the final sample consisted of *N* = 71, is another limitation of the present study, because systematic selection bias could have happened. Most of the drop-out was due to incomplete questionnaires (*n* = 197). Participants with poor relationships with their parents might have terminated the survey, because they simply did not want to share information about their relationship with their parents. Therefore, the final sample might be biased on people who like to share information about their relationship with their parents, which limits generalizability. |
| Statistical power is likely insufficient for the complex moderated mediation models employed. This limitation should be discussed. | This remark follows from the previous point. Implications of the limited statistical power are discussed, and the results are stated more cautiously (line 674 to 679): Finally, the small sample size significantly impacts the statistical power of the moderated mediation model. This has several implications. First, due to the insufficient data small differences could not be detected. Second, the significant association, which was found, might not reflect a true effect. Third, the reproducibility of results might be limited, especially when conducting the study with a more heterogeneous group. Therefore, the present results are only exploratory and have to be replicated in a much larger and more diverse sample. |
| The sample is homogeneous (psychology students, predominantly female, all Caucasian, mean age 21), which substantially restricts the generalizability of the findings. This important limitation should be emphasized. | As added in line 670 to 674, we emphasized to limited generalizability of the present study, due to the low statistical power and the non-representative sample: Another limitation regarding the sample is its homogeneity. The sample mainly consists of Caucasian females about 21 years of age, who study psychology. This is a really narrow population group; therefore, results have to be interpreted with caution. However, Ferber [73], states that a convenience sample is sufficient for preliminary and exploratory studies to get a first overview before spending a lot of money on a representative sample. |
| The Parental Bonding Instrument (PBI) is a valid and widely used tool for assessing retrospective perceptions of parental care and control. However, given the age range of participants (some as old as 48), recall bias is a relevant concern that should be highlighted as a limitation. | This addresses an important point. However, there was only one participant who was 48 years of age, the other participants were between 18 and 28 years of age. Since the PBI is well-established over a 20-year period, the majority of our sample falls into this age. Because of the already small sample size, we decided not to exclude the 48-year-old participant (line 634 to 640). |
| Attachment was assessed exclusively through the self-report ASQ. Since more statistically robust instruments are available, it would be useful to justify the choice of this specific measure. | We are aware that other measures (e.g. AAI) might me more appropriate compared to the ASQ (Jewell et al., 2019). However, since we collect data form our first year students for many years with ASQ to investigate changes in bonding longitudinally, we decided not to “change a winnings horse” in the study at hand. We addressed this at line 625 to 630. |
| Relevant contextual factors (e.g., family structure, SES, life events) that could confound the observed associations were not collected. This omission should be addressed as a limitation. | These important limitations are added in line 643 to 644: Other potential influences, like certain life-events, birth order, or socioeconomic status, should be queried and put into relation. |
| The aggregation of the three CpG sites (-901, -924, -934) into a single composite index (Methyl_3) is not fully justified, especially given that intercorrelations among them were not particularly strong. The rationale for this analytical decision should be clarified. | The reviewer raises an important question, which was addressed in line 349 to 353: Based on the literature, we still aggregated CpG sites -901, -924, and -934 into one variable (Methyl_3), because this cluster is most sensitive to experiences early in life and seems to be responsible for regulation of the OXTR gene expression [11]. So, Methyl_3 was used for further analyses. Additionally, the proposed moderated mediation model was already complex enough when focusing on Methyl_3; therefore, we did not want to calculate three different models, one for each CpG site.  The main rationale behind the aggregation was the literature-based approach to this study. However, future studies should verify the results from Danoff et al. |
| Bonferroni correction was applied only to selected hypotheses. The rationale for this selective application is unclear, and an explicit explanation should be provided. | As stated in line 336 to 339, Bonferroni correction was applied to hypotheses a and b combined as well as to hypotheses c and d combined. The rationale behind the integration is that attachment anxiety and attachment avoidance are correlated. Therefore, only a distinction was made between maternal and paternal care. |
| The discussion is overly long and, at times, digressive. A more concise and focused narrative would improve readability. For instance, certain sections (e.g., lines 454–461) deviate from the main topic and should be reconsidered. | The original structure of the discussion remained the same. However, selected phrases were deleted or rephrased to improve readability and understanding. |
| While the discussion raises interesting points, it tends to justify null results with excessive speculation (e.g., small sample size, retrospective recall, genomic region differences). A more critical and balanced tone is recommended. | As stated above, this is a really important point which was addressed accordingly. A large part of the discussion was reframed and stated more cautiously. |
| Causal language should be avoided, as the cross-sectional design does not allow for causal inference. The phrasing of several statements should be revised accordingly. | Again, the reviewer has a valid point, which we addressed by using a more tempered and more correlational tone, especially in the discussion. |
| The role of paternal care is somewhat downplayed. The absence of significant effects should be acknowledged directly rather than extensively speculated upon. | Thank you for pointing out this shortcoming. We added a large paragraph in line 471 to 489 to address this topic. We talked about different family models and the importance of incorporating those in future studies, because of the way society is changing with respect to caregiving. We added studies which show the perceived differences between mother and father and the assumed consequences. Finally, we shared ideas for future studies. |
| The conclusion should be reframed: the findings provide exploratory evidence rather than definitive support for a mediation/moderation model. | As for the whole discussion, the conclusion was adjusted, especially in line 681 to 685: This exploratory study provides first preliminary insights into the cumulative effect of positive early experiences, DNA methylation in the OXTR MT2 region, and neuroticism on adult attachment. The overall models incorporating anxious and avoidant attachment as well as the distinction between the effect of maternal and paternal care, were not significant. Yet, initial results show that neuroticism significantly moderates the effect of maternal care on OXTR MT2 methylation. |
| Numerical results should generally not be repeated in the discussion, in order to facilitate readability. | No numerical results are repeated in the discussion. The numerical results which are presented are part of further analyses and therefore new to the text. |
| It should be made explicit that the study is exploratory and preliminary in nature. | We revised the whole text accordingly, especially the beginning of the conclusion (line 681 to 682) was framed more cautiously: This exploratory study provides first preliminary insights into the cumulative effect of positive early experiences, DNA methylation in the OXTR MT2 region, and neuroticism on adult attachment. |
| The practical significance of the findings should be discussed more clearly: what contribution do these data make to the field, and what are the implications for future research? | We added a paragraph about the practical significance of the present study in line 690 to 697. Our main point is the focus on personality rather than comparing different childhood experiences, because in our opinion certain personality traits, like neuroticism, can influence certain experiences, which impact their effect. |
| A more measured and cautious tone is recommended in the conclusions, in order to better reflect the exploratory and preliminary nature of the study. | We revised the whole text accordingly, especially the conclusion (line 681 to 697). |
